# From Riluzole to Dexpramipexole via Substituted-Benzothiazole Derivatives for Amyotrophic Lateral Sclerosis Disease Treatment: Case Studies

**DOI:** 10.3390/molecules25153320

**Published:** 2020-07-22

**Authors:** Serge Mignani, Jean-Pierre Majoral, Jean-François Desaphy, Giovanni Lentini

**Affiliations:** 1 Laboratoire de Chimie et de Biochimie Pharmacologiques et Toxicologique, Université Paris Descartes, PRES Sorbonne Paris Cité, CNRS UMR 860, 45, rue des Saints Peres, 75006 Paris, France; 2 CQM-Centro de Química da Madeira, MMRG, Universidade da Madeira, Campus da Penteada, 9020-105 Funchal, Portugal; 3 Laboratoire de Chimie de Coordination du CNRS, 205 route de Narbonne, 31077 Toulouse CEDEX 4, France; jean-pierre.majoral@lcc-toulouse.fr; 4 Université Toulouse, 118 route de Narbonne, 31077 Toulouse CEDEX 4, France; 5 Dipartimento di Scienze Biomediche e Oncologia Umana, Scuola di Medicina, Università degli Studi di Bari Aldo Moro, Piazza Giulio Cesare, 70124 Bari, Italy; jfdesaphy@gmail.com; 6 Dipartimento di Farmacia—Scienze del Farmaco, Università degli Studi di Bari Aldo Moro, via Orabona 4, 70125 Bari, Italy

**Keywords:** riluzole, amyotrophic lateral sclerosis (ALS), dexpramipexole, 1,3-benzothiazole (BTZ), indoles, DrugBank, chronic neurodegenerative disorders

## Abstract

The 1,3-benzothiazole (BTZ) ring may offer a valid option for scaffold-hopping from indole derivatives. Several BTZs have clinically relevant roles, mainly as CNS medicines and diagnostic agents, with riluzole being one of the most famous examples. Riluzole is currently the only approved drug to treat amyotrophic lateral sclerosis (ALS) but its efficacy is marginal. Several clinical studies have demonstrated only limited improvements in survival, without benefits to motor function in patients with ALS. Despite significant clinical trial efforts to understand the genetic, epigenetic, and molecular pathways linked to ALS pathophysiology, therapeutic translation has remained disappointingly slow, probably due to the complexity and the heterogeneity of this disease. Many other drugs to tackle ALS have been tested for 20 years without any success. Dexpramipexole is a BTZ structural analog of riluzole and was a great hope for the treatment of ALS. In this review, as an interesting case study in the development of a new medicine to treat ALS, we present the strategy of the development of dexpramipexole, which was one of the most promising drugs against ALS.

## 1. Introduction

Bicyclic heterocyclic structures are commonly found both in natural and synthetic biologically relevant compounds with indole are probably the most widespread scaffold of this type. In Nature, the indole nucleus may be found in neurotransmitters and autacoids such as serotonin, melatonin, and melanin, which derive from the corresponding amino acid tryptophan, and in a plethora of alkaloids. Dozens of synthetic routes have been developed to afford thousands of compounds based on the indole scaffold [1,2] and more than 300 indole-based small molecules can be found in DrugBank [3]. However, the functionalization of the indole ring may be somewhat limited by its innate nucleophilicity [4], with its relatively high HOMO energy allowing mainly for electrophilic substitution at the 3-position. The same electronic distribution that biases scaffold decoration is the cause of the relative metabolic instability of indole-derived drugs [5] and may confer potential for pan assay interference [6] and host-gut microbiota metabolic interactions [7]. In some instances, the NH hydrogen bond donor group might be unnecessary or even detrimental when binding requirements are to be fulfilled. For all above reasons, scaffold-hopping from indole to a suitable equivalent ring [8] may be advisable, with the 1,3-benzothiazole (BTZ) ring being a relatively less explored alternative [9,10,11,12]. In Table 1, a comparison of the main physicochemical features of the two bicyclic systems is reported and a clear-cut electron density complementarity emphasizes each other’s qualities. Due to its low energy LUMO, BTZ mainly undergoes nucleophilic substitution with the 2-position being preferred, as suggested by its LUMO density distribution. Scaffold-hopping to BTZ should be considered when plasma protein binding is advisable. Plasma protein binding has a strong nonspecific component with the size of drugs playing a major role [13]. Considering both BTZ’s relatively higher metabolic stability and affinity for plasma proteins, the BTZ scaffold should afford longer activity duration.

**Figure 1:**
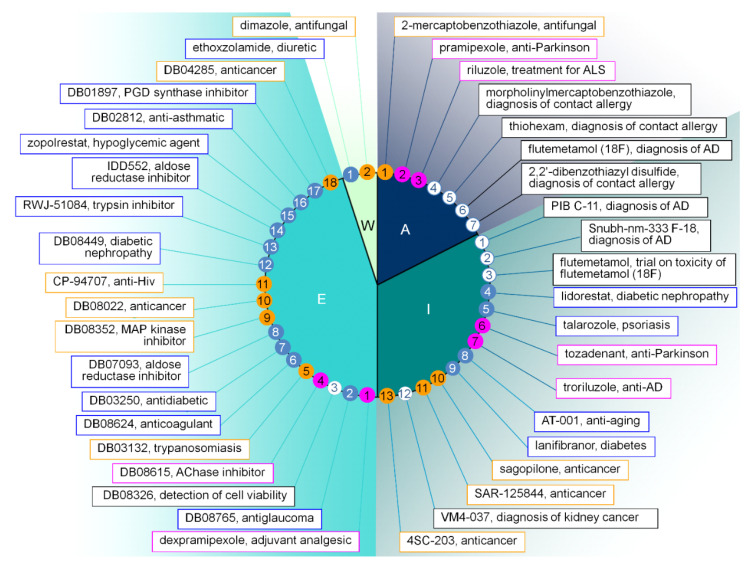
Common names and therapeutic applications of the 1,3-benzothiazole drugs included in DrugBank [3]; A: approved, I: investigational, E: experimental, W: withdrawn; each drug has been color-coded as follows: yellow = etiotropic agent, magenta = drugs acting on CNS, white = diagnostic, and blue = pharmacodynamic agent (see text for details).

**Figure 2:**
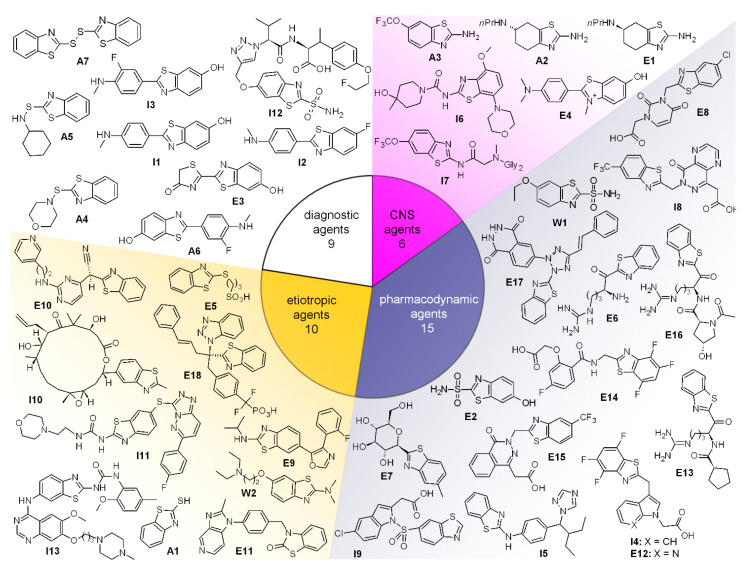
Structures and classes of the 1,3-benzothiazole drugs included in DrugBank^3^; A: approved, I: investigational, E: experimental, W: withdrawn; each therapeutic class has been color-coded as follows: yellow = etiotropic agent, magenta = drugs acting on CNS, white = diagnostic, and blue = pharmacodynamic agent (see text for details).

The substitutions of the BTZ scaffold have shown a broad range of biologic activities, including anti-infective [15], anti-inflammatory [16], antitumor [17], antimyotonic [18], anticonvulsant [19], and neuroprotective properties in both acute and chronic models of neurodegeneration [20]. The approved (A), investigational (I), experimental (E), and withdrawn (W) BTZ derivatives reported in DrugBank [3] are captured in Figure 1 and Figure 2. 

**Figure 3:**
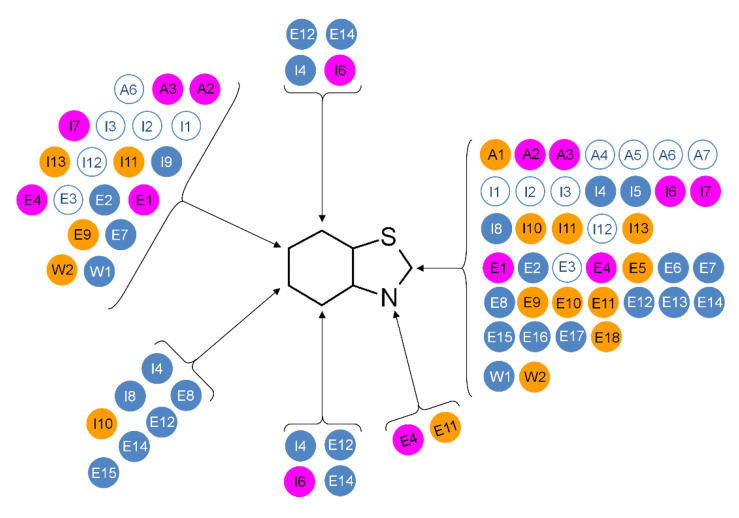
Patterns of substitution displayed by the 1,3-benzothiazole drugs included in DrugBank [3]; A: approved, I: investigational, E: experimental, W: withdrawn; each therapeutic class has been color-coded as follows: yellow = etiotropic agent, magenta = drugs acting on CNS, white = diagnostic, and blue = pharmacodynamic agent (see text for details).

**Figure 4:**
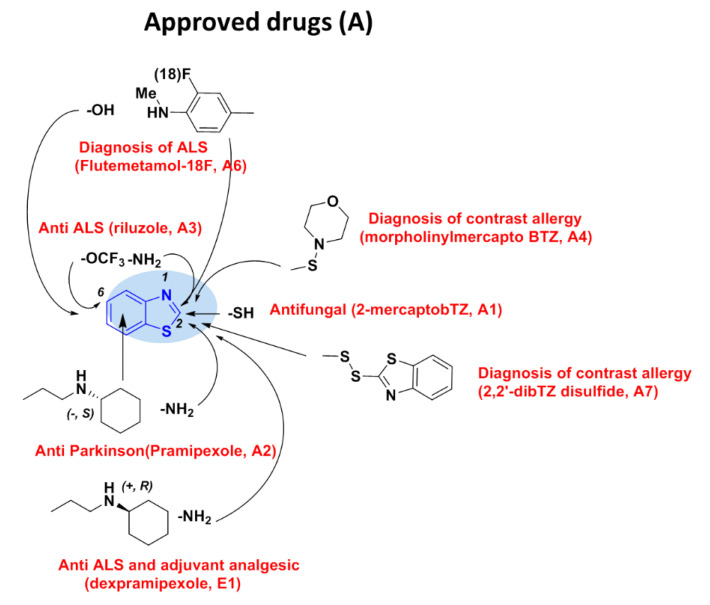
Substituents displayed by the approved 1,3-benzothiazole drugs included in DrugBank [3]; dexpramipexole (E1) was included.

**Figure 5:**
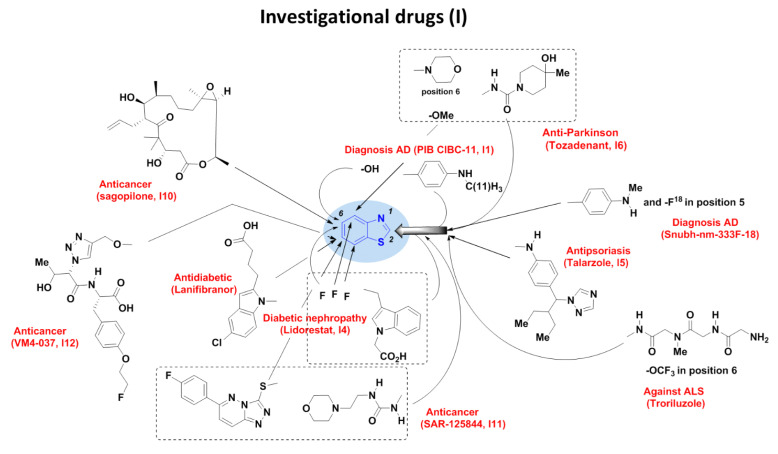
Substituents displayed by the investigational 1,3-benzothiazole drugs included in DrugBank [3].

For the sake of simplicity, four main groups of drugs were considered. Anticancer, antiprotozoal, antifungal, antibacterial, and antiviral activities were generally referred to as etiotropic activities (from the Greek αιτιο, cause, and τροποσ, manner): they share so-called selective toxicity [21] against the unwanted guest (the aggressor) as their basic mechanism with the assumption that cancer cells are no longer considered self-cells. They were color-coded yellow. Both neurological and psychiatric drugs were generally referred to as drugs acting on CNS and magenta is the corresponding color code. They include drugs to alleviate amyotrophic lateral sclerosis (ALS) and anti-Parkinson, anti-Alzheimer, anticonvulsant, analgesic, and antidepressant agents. Diagnostic agents are color-coded white and, finally, all other classes of drugs were included in the general group of pharmacodynamic agents (color-coded blue): they modify autonomic body functions and include antidiabetic, anti-inflammatory, antioxidant, antiallergic, antipsoriasis, and antihypertensive agents. The aforementioned regioselectivity bias which favors substitution onto the 2-position is highlighted in Figure 3. In Figure 4 and Figure 5, more details are given about the substituents found in A and I drugs reported in DrugBank, respectively.

With few exceptions, the BTZ derivatives reported in DrugBank fulfil the requirements for high oral bioavailability (Table 2) [22] with the most interesting compounds being the riluzole (A3) analog pramipexole (A2) and its enantiomer dexpramipexole (E1). A2 and E1 present relatively high 3D structural complexity (they are optically active compounds endowed with a high fraction of sp^3^ carbon atoms) [23] and due to their low molecular weight may be considered as fragments (Ro3 fulfilled).

Despite some contrasting considerations [30,31], high plasma protein binding is generally considered as a factor that possibly limits CNS distribution and reduces potency at that level [32]. CNS penetration decreases as molecular weight increases [33]. The same detrimental effect on blood-brain barrier penetration stems from hydrogen bond acceptor groups [34]. Thus, it might be expected that the BTZ scaffold should be relatively unsuitable for drugs that would act on CNS. Curiously, the most interesting BTZ derivatives—riluzole (A3), pramipexole (A2) and dexpramipexole (E1)—display their activities on the CNS. Probably, an optimized lipophilic balance compromises with features that are detrimental to brain distribution [34]. In this review, we present the strategy of the development of dexpramipexole, which was one of the most promising drugs against ALS and is now under study as an adjuvant analgesic.

## 2. Treatments against Chronic Neurodegenerative Disorders

**Figure 6:**
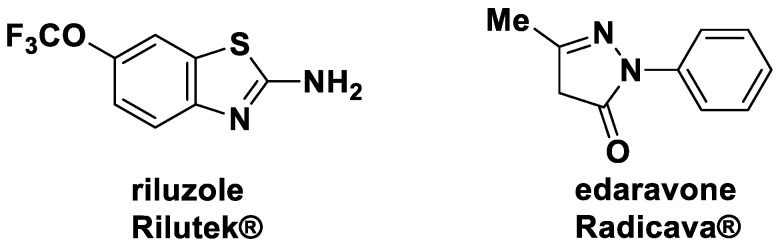
Chemical structure of riluzole (Rilutek^®^) and edaravone (Radicava^®^).

The development of treatments against chronic neurodegenerative disorders such as Alzheimer’s (AD) and Parkinson’s (PD) diseases, as well as amyotrophic lateral sclerosis (ALS; also known as Lou Gehrig’s disease or Charcot’s sclerosis), and acute conditions such as brain ischemia and trauma have proven extremely difficult. Among others, these diseases are connected with the excitotoxicity of excitatory amino acids (EAAs) as neurotransmitters [35]. Excessive release of glutamate and a subsequent over activation of excitatory amino acid receptors such as *N*-methyl-d-aspartate (NMDA), α-amino-3-hydroxy-5-methyl-4-isoxazolepropionic acid (AMPA), and ainite receptors represent the main steps of the excitotoxic cascade. These attractive targets have been studied for the development of neuroprotective agents. A very interesting class of neuroprotective agents is represented by a simple benzothiazole ring as a scaffold. One of them is riluzole (Rilutek^®^, 2-amino-6-(trifluoromethoxy)benzothiazole, Figure 6), which has demonstrated neuroprotective effects in several animal models of PD [36], Huntington’s disease [37] and cerebral ischemia [38]. Riluzole is currently the only Food and Drug Administration (FDA)-approved drug for the treatment of ALS. The drug prolongs survival time by a few months and delays the use of invasive supportive therapies, such as mechanical ventilation and tracheotomy, but shows little efficacy on muscle strength or function [39,40,41,42,43]. Riluzole is a nonspecific neuroprotectant.

Radicava® (edaravone, Figure 6), with antioxidant properties, was approved for the treatment of ALS in Japan (2015) and in the United States (2017). In 2015, the drug was designated an orphan medicine for ALS by the European Medicine Agency. However, on 24 May 2019, Mitsubishi Tanabe Pharma GmbH formally notified the Committee for Medicinal Products for Human Use (CHMP) of its wish to withdraw its marketing authorization application for the treatment of ALS due to unfavorable benefit risk balance [44]. This withdrawal did not have impact on the ongoing clinical trials with Radicava^®^. The chemical structure of edaravone (5-methyl-2-phenyl-2,4-dihydro-3*H*-pyrazol-3-one) is different from that of riluzole.

To date, the causes of ALS, both familial (~10%) and sporadic (~90%), are unknown, except for a small number of inherited monogenic cases. ALS is universally fatal, characterized by progressive weakness [45] due to the degeneration of upper and lower spinal motor neurons, and leads eventually to respiratory failure, which is the usual cause of death. The major proposed mechanisms of ALS are glutamate excitotoxicity, oxidative stress, mitochondrial dysfunction, protein aggregation, superoxide dismutase 1 (SOD1) accumulation and neuronal death [46].

In early clinical trials with 876 ALS patients receiving riluzole compared to 406 patients receiving placebo, riluzole showed an increase in mean survival time, varying from 3 months (dose, 100 mg/day) to 1.7 months (various dose levels) [41].

Riluzole was developed by the Pharmuka Laboratoires (Antony, France) in the 1980s under the reference PK 26124. Pharmuka Laboratoires became Rhône-Poulenc Rorer (Antony, France) in 1983, now named Sanofi. Riluzole was found using a phenotypic screening strategy and was shown to be capable of interfering with the mechanisms of neuronal death in the ALS. Currently, the drug is marketed by Sanofi under the brand name Rilutek^®^. In 2012, it became available as a generic drug.

Riluzole was first tested in the early 1980s as an antiepileptic, based for example, on its action to prevent rodent convulsions induced by electroshock, γ-aminobutyric acid (GABA) synthesis inhibitors, or ouabain. This anticonvulsant activity was not due to an effect on GABAergic neurotransmission, as with other classical treatments, but relied on an antagonistic action against neurotransmission by certain excitatory amino acids. A few years later, riluzole was included in experimental models broader than those just for epilepsy, such as ischemia. It was shown that, as expected from its antiglutamatergic activity, riluzole decreased neuronal death due to ischemia. This antiglutamatergic action appeared quite peculiar, because riluzole does not bind to any of the known excitatory amino acid receptors but appears to act primarily at the presynaptic level, blocking the release of glutamate. The results of the first clinical trial for the treatment of ALS by riluzole, involving 155 patients and carried out in double-blind, showed an effect on patient survival. The data was even better for the bulbar form of ALS, which is rarer.

Generally speaking, several speculative mechanisms of action of riluzole have been proposed, including the inhibition of voltage-gated sodium channels, which can reduce neurotransmitter release, noncompetitive inhibition of NMDA receptors [47], inhibition of glutamate release [48], and enhanced astrocytic uptake of extracellular glutamate [49]. Experiments with animal models of excitotoxicity and injury demonstrated that riluzole plays an important role as a neuroprotective agent [50]. In addition, riluzole is known also to be an antioxidant agent [51], an antiapoptotic agent [51] and has beneficial effects on neuronal cell death due to cisplatin-induced ototoxicity [52].

There are an increasing number of patients diagnosed with ALS with an incidence between 0.6 and 3.8 per 100,000 people/year in the World. The incidence is higher in Europe, between 2.1 and 3.8 per 100,000 people/year. Recent studies have reported a prevalence of ALS between 4.1 and 8.4 per 100,000 people. In addition, a difference in ALS prevalence by ethnicity was reported in the United States [53]. Consequently, the need to find new alternatives to the current single treatment using riluzole is becoming increasingly urgent in order to treat ALS-affected patients.

The purpose of this manuscript is to review the development of dexpramipexole for the treatment of ALS. It should be noted that this structural analog of riluzole, was not synthesized to mimic the efficacy of riluzole in ALS by exploiting its structural similarity [54]. In this respect, the origin of dexpramipexole development differs from the one of the monosubstituted 2-benzo-thiazolamines and 3-substituted 2-iminobenzothiazoline derivatives developed by Rhône-Poulenc Rorer (Vitry-sur-Seine center, Vitry-sur-Seine, France) [55] and, recently, by Sweeney et al. from the University of Bradford (Bradford, UK) [56]. To the best of our knowledge, two drug therapies have been developed in the clinical phase to fight ALS, the FDA-approved riluzole and dexpramipexole, which represents an interesting case study in that is chemically close to riluzole. Riluzole and its SARs were mainly developed in Rhône-Poulenc/Aventis in Vitry-sur-Seine research center (France). Other potential drugs against ALS are the hydroxylamine arimoclomol [57], the noncompetitive AMPA agonist perampanel [58], the beta-lactam antibiotic ceftriaxone [59], the free-radical scavenger bromocriptine [60], and the non-steroidal anti-inflammatory nimesulide [61]. Other strategies to tackle ALS are stem cell therapy [62] and immunotherapy [63].

## 3. 6-Substituted 2-Benzothiazolamines and 3-Substituted 2-Iminobenzothiazoline Derivatives as Analogues of Riluzole

**Figure 7:**
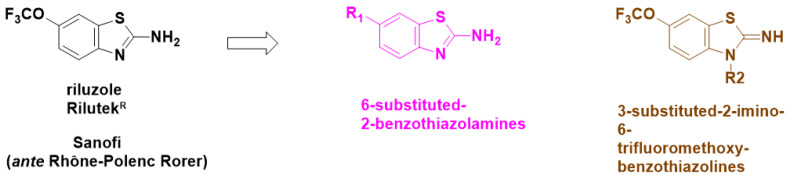
Chemical structure of 6-substituted-2-benzothiazolamines and 3-substituted-2-iminobenzothiazolines.

In the 1950s, the major studies regarding the development of 2-benzothiazolamines were dedicated to central muscle relaxants [64]. Then, riluzole had shown interesting neuroprotective slowing ALS progression in clinical trials (*vide supra*). Taking advantages of these important studies, two series of close analogues of riluzole were prepared by Mignani’s teams in Rhône-Poulenc Rorer in the 90s: 6-substituted 2-benzothiazolamines and 3-substituted 2-iminobenzothiazolines (Figure 7) [55]. All of these riluzole analogues were tested in vivo (ip administration) for the protection against glutamic acid evoked convulsions in rats as a model of neuroprotection. One advantage of this in vivo model is that it takes account of the anticonvulsant effects related to the bioavailability of each compound.

**Scheme 1:**
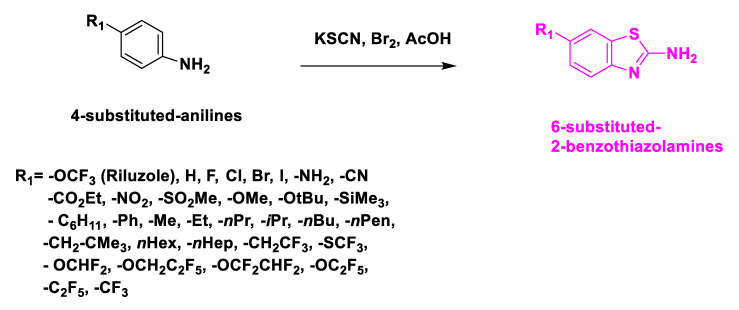
Synthesis of 6-substituted 2-benzothiazolamines.

More than 30 6-substituted 2-benzothiazolamines were prepared by one-pot reaction of the corresponding aniline with thiocyanogen generated from bromine and alkaline thiocyanate (Scheme 1) in good yield (> 50%).

**Scheme 2:**
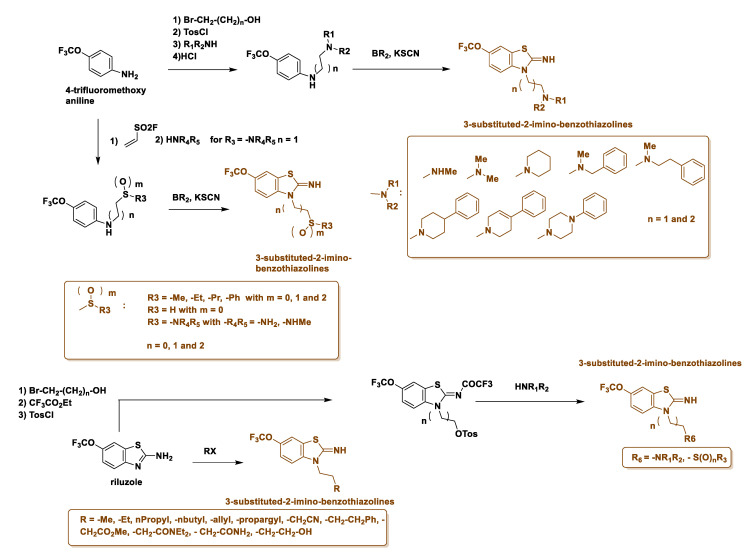
3-Substituted 2-iminobenzothiazolines.

More than 40 3-substituted 2-imino benzothiazolines were prepared. As shown in Scheme 2, several synthetic pathways were developed, two starting from 4-trifluoromethoxyaniline and two from riluzole, in moderate to good overall yields [55].

**Figure 8:**
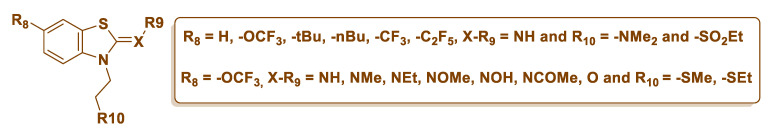
Simultaneous modifications in positions 3 and 6, and 2 and 3.

In addition, a small library of simultaneous modifications in positions 3 and 6, and 2 and 3 were prepared using the synthetic pathways described in Scheme 1 and Scheme 2 (Figure 8) [55].

All of the prepared riluzole analogues were evaluated in two models of neuroprotection: protection against seizures induced by intracerebroventicular administration of glutamic acid in rats and protection against mortality induced by hypobaric hypoxia in mice [55]. The most effective compounds in these models are presented in Table 3.

A significantly enhanced antiglutamatergic activity was obtained by the introduction of sulfur- or nitrogen-containing 3-substituents. The most potent derivatives were 2-imino-3-(2-methylthio)- and 2-imino-3-(2-methylsulfinyl)ethyl-6-trifluoromethoxybenzothiazolines (**II** and **III**, respectively). The introduction of piperidine (**V**), 4-phenylpiperidine (**VI**), 4-phenyl-1,2,3,6-tetrahydropyridine (**VII**) and 4-phenylpiperazine (**VIII**) linked with an ethyl chain in position 3 afforded potent neuroprotective compounds similar to riluzole.

In 2007, Gunakkunru and Verma described interesting studies about the quantitative structure-activity relationship of riluzole series from the Mignani’s team results (*vide supra*) [65]. From 24 riluzole analogues (2-benzothiazolamines and 3-substituted-2-imino benzothiazolines), QSAR models were developed. The descriptors used in this study were: polarizability, density, molar refractivity, molar volume, average mass, and parachor, as well regression data and cross validation parameters. The 3-substituted iminobenzothiazolines can be modeled remarkably using index of refraction, surface tension, and average mass.

**Figure 9:**
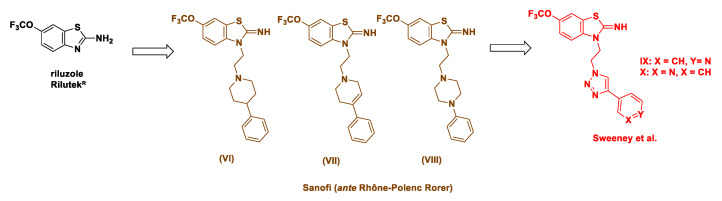
Chemical structures of the most potent hybrids of riluzole (**VI**, **VII**, **VIII**, **IX** and **X**).

*Post etiam*, in 2018, Sweeney et al. described the synthesis of triazole-containing riluzole analogues and their neuroprotective effects [56]. The 49 compounds prepared were chemically close to the 6-substituted-2-benzothiazolamines (e.g., **VI**, **VII** and **VIII**) described in Figure 9, and were prepared using click reaction. Within this library, seven compounds, excluding riluzole, attenuated kainate-induced neurofilament loss, and together with riluzole resulted positive in the MAPS assay in primary cortical neurons. The compounds **IX** and **X** (Figure 9) displayed the greatest effect.

## 4. Pifithrin-α with Neuroprotective Properties

**Scheme 3:**
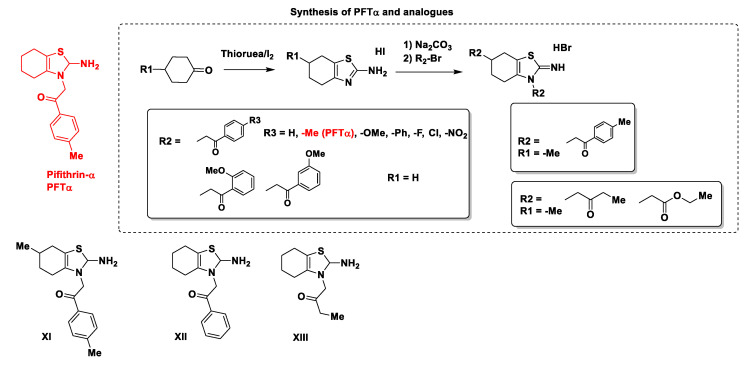
Chemical structures of pifithrin-α, **XI, XII, XII** and their synthetic pathway.

Pifithrin (PFT)-α contains a partially saturated bicyclic thiazolyl core (Scheme 3). It is a p53 inhibitor preventing neuronal cell death by inhibiting p53 transcriptional activity, mitochondrial damage, and caspase activation. PTFα showed neuroprotective effects against traumatic brain injury in the striatum through suppression of neuroinflammation, oxidative stress, autophagy, and apoptosis [66]. PTFα was effective for the neuronal cell protection reducing the side effects of anticancer drugs. Zhu et al. showed that PTFα and several close analogues (**XI**, **XII** and **XIII**) exhibited neuroprotective activity in tissue cultures such as in PC12 and hippocampal cells from camptothecin-induced cell death [66]. In vivo, PTF displayed neuroprotection in transient and permanent stroke mouse models at the dose of 2 mg/kg ip.

## 5. Dexpramipexole for the Treatment of ALS

**Figure 10:**
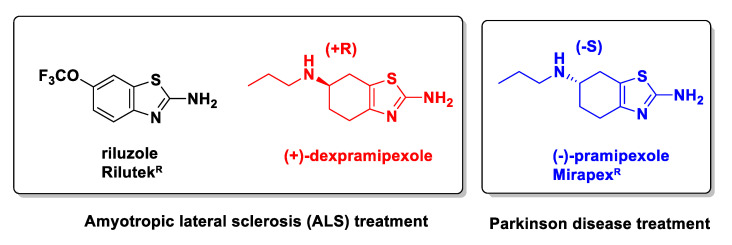
Chemical structures of riluzole, dexpramipexole, and (−)-pramipexole.

The benzothiazolamine (–)-pramipexole (*S*-configuration, Mirapex^®^; also named PPX) is a medication indicated for the treatment of Parkinson’s disease and the restless legs syndrome. PPX enantiomer dexpramipexole [KNS-760704, (6*R*)-(+)-4,5,6,7-tetrahydro-*N*-propyl-2,6-benzo-thiazolediamine dihydrochloride; also named BIIB 050 or RPPX] has been found to be a good neuroprotective agent in vitro and in several animal models, and may therefore be of interest for the treatment of ALS (Figure 10) [67].

Dexpramipexole was first recognized as a potential drug against ALS by several groups such as Ferrari-Toninelli et al. [68], who described that both (–)-pramipexole and dexpramipexole showed antioxidant and neuroprotective activity, independent of chirality using neuroblastoma cells. Equipotent efficacy in preventing cell death induced by H_2_O_2_ and inhibiting mitochondrial reactive oxygen species generation were observed.

Dexpramipexole is a weak nonergoline dopamine agonist, and unlike its *S*-isomer (–)-pramipexole, displays agonist activity on dopamine D2, D3, and D4 receptors. It also exhibited significant neuroprotective properties independent of its dopamine receptor agonism [69]. Very recently and similarly to riluzole, it was shown to inhibit voltage-gated Nav1.8 sodium channels and provide analgesia in various nociceptive and neuropathic pain models [70,71]. (–)-Pramipexole (Mirapex^®^) was developed by Boehringer Ingelheim (Ridgefield, CT, USA) and was approved by the US FDA in 1997 and by the European Medicines Agency (EMA) in 1998. (–)-Pramipexole is used orally for the treatment of idiopathic Parkinson’s disease and represents a novel class of nonergot selective dopamine receptor agonists. Mirapex® is used as monotherapy and as adjunctive therapy with levodopa [72]. An interesting study has been described by Javan et al. regarding the in vitro long-acting formulation for (–)-pramipexole based on poly(3-hydroxybutyrate-co-3-hydroxy-valerate) nanoparticles. (–)-Pramipexole is included inside the matrix of the polymer, and it is gradually released within 30 days [73]. In addition, several studies highlighted the therapeutic use of (–)-pramipexole for the treatment of cognitive disorders [74].

An interesting study regarding the repurposing of dexpramipexole in neonatal hypoxic/ischemic encephalopathy has been highlighted. It was shown that dexpramipexole at the dose of 3 mg/kg (b.i.d., ip), after the hypoxic insult due to distal middle cerebral artery occlusion, decreased the infarction size in pups with mild to moderate injuries [75].

Compared to the *S*-isomer (–)-pramipexole, for which stereospecific dopamine receptor affinity difference was observed, dexpramipexole has a much lower dopamine agonist activity. Nevertheless, using nondopaminergic JK cells in cell-based assays, similar specific biological effects were observed for the two enantiomers: both: 1) reduced the production of reactive oxygen species (ROS), 2) decreased the activation of apoptotic pathways, and 3) increased cell survival in response to a variety of neurotoxins such as glutamate. Both enantiomers were shown to enter the brain with a brain-to-plasma ratio in excess of 6 in mice. At a lower dose, (–)-pramipexole administration was associated with a significantly increased wheel-running behavior attributed to dopaminergic activation. Consequently, (–)-pramipexole has a limited clinical utility as a neuroprotective agent due to its unacceptable dopaminergic side effects, including hypotension and seizures, but is currently used to treat symptoms of Parkinson’s disease. Unlike (–)-pramipexole, dexpramipexole that displays similar neuroprotective activity but limited dopaminergic agonism, could be used in the treatment of ALS. The exact mode of action of dexpramipexole is unknown, but it showed a neuroprotective effect on neurons under stress within motor neuron cells [76].

Importantly, the lack of significant D2 dopamine receptor activation of dexpramipexole could allow for theoretically reaching much higher drug levels in CNS. Physicochemical, pharmacokinetic (PK/PD), and toxicokinetic profiles of dexpramipexole in preclinical studies were performed. Dexpramipexole is highly water-soluble (> 600 mg/mL), highly stable in solution in water as well in physiological buffer solutions, and it is not hygroscopic. Dexpramipexole is neither mutagenic nor genotoxic, did not significantly affect the cardiac delayed rectifier (hERG) potassium current, and in a good laboratory practices (GLP) study, showed good cardiovascular safety in Gottingen minipigs. No adverse effects were observed at the highest dose tested (75 mg/kg). In order to get IND approval, acute and chronic toxicology studies have been fully completed (in rats and minipigs) and clearly showed that dexpramipexole could be developed for ALS treatment.

**Scheme 4:**
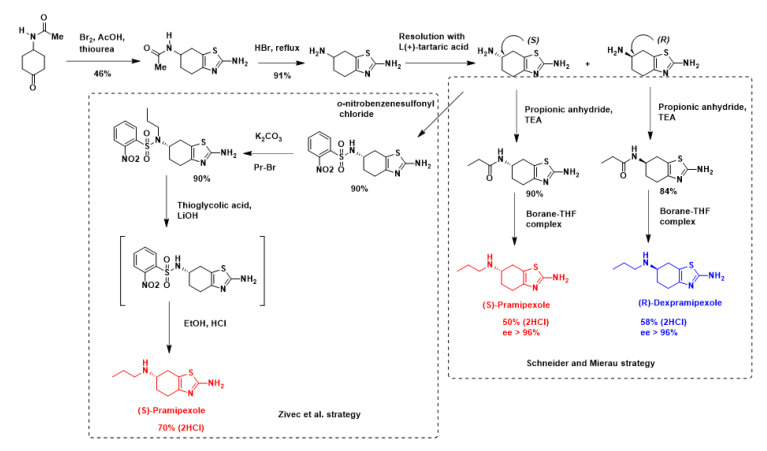
Synthesis of (*S*)-pramipexole and dexpramipexole using the optical resolution strategy.

The preparation of (–)-pramipexole occurred via the resolution of racemic (±)-pramipexole [77] or using preparative chiral chromatography to separate (–)-pramipexole and dexpramipexole [78], but these processes are long and tedious. Consequently, an asymmetric synthesis was performed. The scalable synthetic pathway and resolution of (–)-pramipexole was presented by Zivec et al. [79] as an industrially acceptable process and also, previously, by Schneider and Mierau (Scheme 4) [80]. (–)-Pramipexole was first described in the European patent application EP 0186087.

**Scheme 5:**
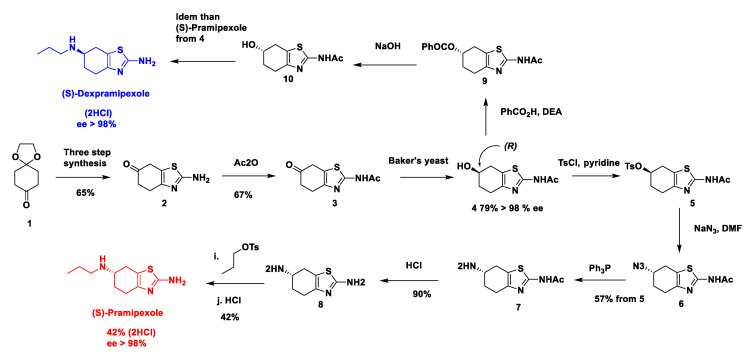
Synthesis of (–)-pramipexole and dexpramipexole using the biocatalyst strategy.

Interestingly, Ferraboschi et al. described the preparation of (–)-pramipexole and dexpramipexole using baker’s yeast based on the highly stereoselective transformation allowed by these biocatalysts (Scheme 5) [81].

### 5.1. Clinical Trials of Dexpramipexole for the Treatment of ALS

Dexpramipexole has been investigated in clinical trials for ALS by Knopp Biosciences (Pittsburgh, PA, USA) and Biogen Idec (Cambridge, MA, USA). In 2004, dexpramipexole was introduced into phase I based on new drug application acceptance (IND 60,948) by James Bennett at the University of Virginia (Charlottesville, VA, USA). Fifteen ALS patients received escalated dosing from 1.5 to 30 mg/day, where no complications were observed, and then continued to 30 mg/day for an additional eight weeks. Based on these results, clinical efficacy studies of the 30 mg/day dose were started in September 2005, and the dose-escalation study from 30 to 300 mg/day was initiated in January 2006. In September 2009, the FDA gave a fast track designation for the development of dexpramipexole for ALS [82], and it has received orphan drug designation by both the FDA and the EMA.

Based on the excellent tolerability of higher doses of dexpramipexole (phase I), initial phase II dosing experiments were advanced. In a futility design study, the dose of 30 mg/day of dexpramipexole was orally administrated (*n* = 30 patients) to evaluate its therapeutic efficacy. Analysis of the data showed individual declines in the standard measure of disability in ALS, named the ALS functional rating scale (ALSFRS-R) and neurophysiological index (NI) values, but with nonsignificant reductions during treatment. Consequently, dosing with 30 mg/day of dexpramipexole did not induce clinically meaningful effects on ALS progression. Then, in a dose-escalation study, excellent safety and tolerability after oral administration were also observed up to a maximum daily dose of 100 mg three times a day (TID) without dopaminergic side-effects (e.g., discernible effects, QTc intervals). The trough and peak of the drug increased linearly with dosing (30 to 300 mg/day dose-escalation study). Observation of 60 mg/day induced a small slowing of decline in ALSFRS-R scores but was not significant in the small population included in the clinical studies. Excellent safety and tolerability of oral administration of dexpramipexole up to 300 mg/day were observed based on one year of cumulative dosing, as well as extensive distribution into body tissue stores; there was statistically nonsignificant improvements at 30 and 60 mg/day dosing. These results supported the long-term testing of higher dexpramipexole doses to treat ALS [83].

In order to confirm safety at a range of doses and to determine if there was still an efficacy signal with double-blind treatment, a phase II clinical development of dexpramipexole in subjects with ALS was also advanced in two different and consecutive parts (1 and 2), double-blind safety and tolerability studies based on its effects on the decrease of the disease and mortality for dose selection studies. Dr. Cudkowicz and colleagues directed these clinical studies from the Northeast ALS Consortium, a nationwide ALS clinical trial group [84]. In part 1, dexpramipexole was administered 50, 150 or 300 mg/day orally every 12 h vs. randomized placebo over 12 weeks (n = 102 newly diagnosed ALS patients, familial or sporadic ALS, in 20 US sites). In part 2, patients who completed all 12 weeks of part 1 were eligible for randomization in part 2, after a 4-week, single-blind placebo washout strategy, followed by randomized, double-blind, active treatment for 24 weeks (50 mg/day or 300 mg/day). The primary outcome was the safety, whereas the secondary outcomes were safety, the changes in ALSFRS-R, vital capacity, cystatin C, and neurofilament H. Generally speaking, dexpramipexole was safe and well-tolerated. Dose-dependent attenuation of the slope of the decline of ALS was observed in part 1, and a significant difference between groups and mortality was observed in part 2.

In summary, in phase II trials dexpramipexole (25 to 150 mg b.i.d.) was well tolerated for up to 9 months and showed a significant benefit at the high dose in a combined assessment of function and mortality in patients with amyotrophic lateral sclerosis.

Dexpramipexole was then investigated by Knopp Biosciences and Biogen Idec for the treatment of ALS in a randomized, double-blind, phase III trial. The objective of this study was to evaluate whether dexpramipexole (oral tablet, 150 mg twice daily for up to 18 months) was safe and effective in the treatment of ALS. In 2011, two clinical trials were started with a total of 1,549 patients (familial or sporadic ALS) in multi-international centers. Dexpramipexole was generally well tolerated but did not differ from placebo on any specified efficacy endpoint measurement. The drug failed to show efficacy in terms of function or survival of patients with ALS.

### 5.2. Recent Additional in vivo Studies of Dexpramipexole for the Treatment of ALS

In 2014, Vieira et al. reported the non-effectiveness of dexpramipexole in two in vivo models of ALS-related neurodegeneration [72]. In the past two decades, important advances have been made in the identification of genes that predispose individuals to develop inherited ALS; for instance: 1) SOD1 was the first gene to be identified as causing ALS when mutated; 2) identification of neuronal cytoplasmic 43 kDa Tar DNA binding protein (TDP43). This protein is a prominent pathological hallmark in both familial and sporadic ALS; mutations in the gene were identified in familial cases of ALS. The two in vivo parallel screening systems were based on: 1) sibling-matched, gender-balanced survival efficacy screening in high-copy B6-SJL-SOD1G93A/Gur1 mice, and 2) neuronal survival screening in primary rat cortical neurons transfected with wildtype human TDP43 or mutant human TDP43. Dexpramipexole was administered approximatively at the levels used in the phase II clinical trial. No difference between the control and experimental conditions was observed in both in vivo studies.

### 5.3. Clinical Trials of Dexpramipexole for the Treatment of Asthma

In addition to the research on ALS, dexpramipexole also was found to significantly decrease absolute eosinophil counts (AECs), and this opened up opportunities to use it against hypereosinophilic syndromes (HESs) [85]. The HESs are a heterogeneous group of disorders characterized by peripheral eosinophilia and eosinophil-related end-organ damage. Interestingly, as a proof-of-concept study, dexpramipexole was evaluated in a phase II open-label trial and showed a reduced eosinophil count and eliminated the need for corticosteroid treatment in a subset of patients with steroid-responsive HESs. Dexpramipexole was orally administered twice daily (150 mg). Bone marrow biopsy samples after 12 weeks of dexpramipexole showed selective absence of mature eosinophils in responders. Dexpramipexole appeared promising as a glucocorticoid (GC)-sparing agent without apparent toxicity in a subset of subjects with GC-responsive HESs. Knopp Biosciences is advancing the drug in both HESs and eosinophilic asthma [86].

## 6. Conclusions and Perspectives

Efforts to uncover better treatments to slow down, stop, or reverse neurodegeneration in ALS continue to be considered to treat this challenging disease; however no effective treatment is currently available. Research into ALS has been marked by successive failures in all clinical trials since riluzole was first marketed. In this review, we analyzed the development of dexpramipexole, including clinical trials as well the analogues of riluzole (substituted-benzothiazole derivatives) and Pifithrin-α as neuroprotectors. This analysis represents an interesting case study for a scientist working in the CNS domain in general and neuroprotection in particular, such as ALS. In light of the favorable safety profile of dexpramipexole in humans, this drug might have a realistic translational potential to treat other disorders, including other rare diseases.

**Figure 11:**
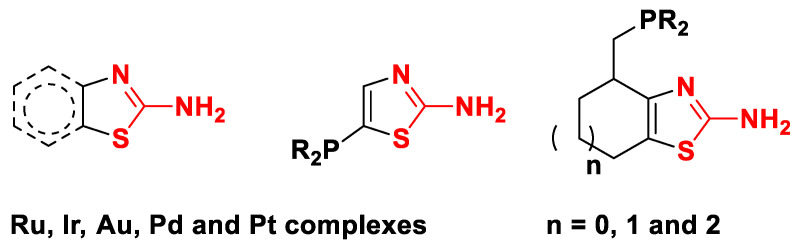
Chemical structures of modified benzothiazolamines.

Due to the key role played by several modified benzothiazolamines, their corresponding low molecular weight, and the importance of the nature of the substituents grafted on the benzothiazole structure, it might be of great interest to prepare the corresponding benzothiazole substituted at different positions of their backbone. For example, the introduction of phosphonate or phosphino groups may be envisaged, and this in turns would allow to prepare different complexes (e.g., Au, Ru, Ir, Pd) with the possibility to enhance the activity of benzothiazolamines or to open new perspectives in medicinal chemistry (Figure 11) [87,88].

It is also worth to note that the variability of the clinical symptoms and biological factors of this pathology justify the development of a personalized therapeutic approach for future clinical trials in the ALS field. In addition, in the personalized medicine field to treat patients with ALS, data from the literature suggest that there is a metabolic defect in ALS. This alteration corresponds to an increase in metabolism (the hypermetabolic pathway), and this dysfunction concerns the metabolism of lipids and carbohydrates, as well as disturbances in the mitochondrial function. Analysis of the metabolic profile of each patient with ALS should be an opportunity to tackle ALS based on personalized medicine, a point highlighted by Vandoorne et al. [89]. Following this direction, based on international collaborative efforts, population-based studies are clearly needed to gain a better estimate of the ALS burden, and as well, the understanding of ALS risk factors. The development of drugs based on metabolic approaches is a hope for treating this disease.

Finally, 2-aminobenzothiazole derivatives may be exploited as fragments in fragment-based approach strategy to find new active agents as described recently by Alnabulsi et al. in the discovery of amino-carboxamide benzothiazoles as potential lysine specific demethylase enzyme LSD1 inhibitors [90].

## Figures and Tables

**Table 1:** Main physicochemical features of indole and 1,3-benzothiazole rings.

Property	Indole	1,3-Benzothiazole (BTZ)
Structure	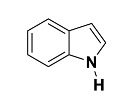	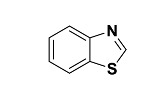
Composition	C_8_H_7_N	C_7_H_5_NS
fw	117	135
Electrostatic potential map ^1,2^	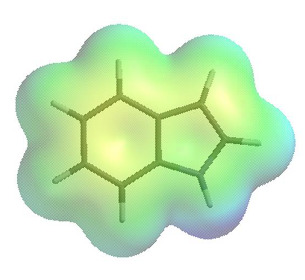	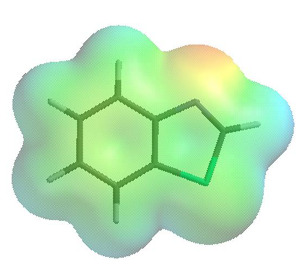
HOMO map ^1^	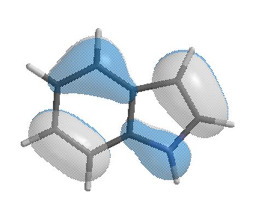	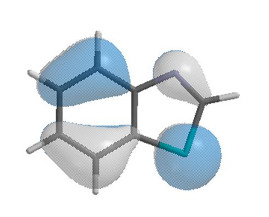
HOMO energy ^1^	−5.81 eV	−6.75 eV
LUMO map ^1^	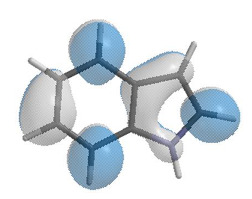	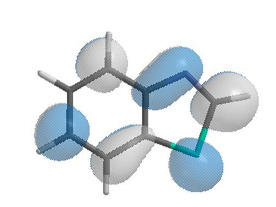
LUMO energy ^1^	−0.61 eV	−1.38 eV
HBA ^3^	-	1
HBD ^4^	1	-
cLog *P* ^5^	2.14	2.01

^1^ Calculated at the DFT B3LYP/6-311+G(2df,2p)//DFT B3LYP/6-311+G(2df,2p) level [14]. ^2^ Warm colors indicate relatively high electron density with red color corresponding to the highest electron density regions; cold colors indicate relatively low electron density with blue color corresponding to the lowest electron density regions. ^3^ HBA, hydrogen bond acceptor. ^4^ HBD, hydrogen bond donor. ^5^ ACD/Labs 7.0.

**Table 2:** Good-quality criteria applied to 1,3-benzothiazole drugs included in DrugBank [3].

Identifier	Class	Ro5 ^a^	Veber Rule ^b^	Ghose Filter ^c^	MDDR Rule ^d^	Ro3 ^e^	Fsp^3 f^	Chirality
**A1**	etiotropic	yes	yes	yes	no	yes	no	no
**A2**	CNS agent	yes	yes	yes	no	yes	yes	yes
**A3**	CNS agent	yes	yes	yes	no	yes	no	no
**A4**	diagnostic	yes	yes	yes	no	yes	yes	no
**A5**	diagnostic	yes	yes	yes	no	no	yes	no
**A6**	diagnostic	yes	yes	yes	no	no	no	no
**A7**	diagnostic	no	yes	no	no	no	no	no
**I1**	diagnostic	yes	yes	yes	no	no	no	no
**I2**	diagnostic	yes	yes	yes	no	no	no	no
**I3**	diagnostic	yes	yes	yes	no	no	no	no
**I4**	pharmacodynamic	yes	yes	yes	no	no	no	no
**I5**	pharmacodynamic	no	yes	yes	yes	no	yes	yes
**I6**	CNS agent	yes	yes	yes	no	no	yes	no
**I7**	CNS agent	yes	yes	yes	no	no	yes	no
**I8**	pharmacodynamic	yes	yes	yes	no	no	no	no
**I9**	pharmacodynamic	yes	yes	yes	no	no	no	no
**I10**	etiotropic	no	no	no	no	no	yes	yes
**I11**	etiotropic	no	yes	no	yes	no	yes	no
**I12**	diagnostic	no	no	no	yes	no	yes	yes
**I13**	etiotropic	no	no	no	yes	no	yes	no
**E1**	CNS agents	yes	yes	yes	no	yes	yes	yes
**E2**	pharmacodynamic	yes	yes	yes	no	no	no	no
**E3**	diagnostic	yes	yes	yes	no	no	no	no
**E4**	CNS agent	yes	yes	yes	no	no	no	no
**E5**	etiotropic	yes	yes	yes	no	no	yes	no
**E6**	pharmacodynamic	yes	yes	yes	no	no	yes	yes
**E7**	pharmacodynamic	yes	yes	yes	no	no	yes	yes
**E8**	pharmacodynamic	yes	yes	yes	no	no	no	no
**E9**	etiotropic	yes	yes	yes	no	no	no	no
**E10**	etiotropic	yes	yes	yes	no	no	no	yes
**E11**	etiotropic	yes	yes	yes	no	no	no	no
**E12**	pharmacodynamic	yes	yes	yes	no	no	no	no
**E13**	pharmacodynamic	yes	yes	yes	no	no	yes	yes
**E14**	pharmacodynamic	yes	yes	yes	no	no	no	no
**E15**	pharmacodynamic	yes	yes	yes	no	no	no	no
**E16**	pharmacodynamic	yes	no	no	no	no	yes	yes
**E17**	pharmacodynamic	yes	yes	no	yes	no	no	no
**E18**	etiotropic	no	yes	no	yes	no	no	yes
**W1**	pharmacodynamic	yes	yes	yes	no	no	yes	no
**W2**	etiotropic	yes	yes	yes	no	no	yes	no

^a^ Rule of Five: hydrogen bond donors (HBDs) ≤ 5, hydrogen bond acceptors (HBAs) ≤ 10, MW ≤ 500 Da, cLog *P* ≤ 5 [24]; b polar surface area (PSA) ≤ 140 Å^2^, rotatable bonds (RBs) ≤ 10, HBDs + HBAs ≤ 12 [25]; ^c^ −0.4 < cLog*P* < 5.6, 160 < MW < 480, 40 < molar refractivity (MR) < 130, 20 < number of atoms < 70 [26]; ^d^ MACCS-II Drug Data Report (MDDR)-like rule: ring number (RN) ≥ 3, RBs ≥ 6, rigid bonds (RBs) ≥ 18 [27]; ^e^ Rule of Three: hydrogen bond donors (HBDs) ≤ 3, hydrogen bond acceptors (HBAs) ≤ 3, MW < 300 Da, cLog*P* ≤ 3 [28]; ^f^ fraction of sp^3^ hybridized carbon atoms (i.e., number of sp^3^ hybridized carbons/total carbon count) > 0.2 [29].

**Table 3:** Neuroprotective activities of benzothiazolines.

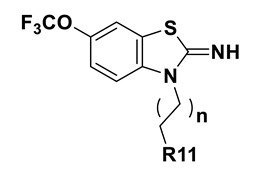				
Compound	-R_11_	n	Glu ^a^	H.H. ^b^
			ED_50_ (mg/kg ip)	
**I**	**-SMe**	0	1.0	14.0
**II**	**-SOMe**	1	**1.1**	**5.5**
**III**	**-SO_2_Me**	1	**1.8**	**2.5–10**
**IV**	**-NMe_2_**	1	2.3	4.0
**V**		1	2.0	<5
**VI**		1	3.0	2.5–10
**VII**		1	2.2	2.5–10
**VIII**		1	3.5	5.5
**riluzole**			3.2	4.0

^a^ Protection against glutamic acid-evoked convulsions in rats. Variability: 15 and 25%. ^b^ Protective effect against mortality induced by hypobaric hypoxia in mice. Variability: 20%.
